# A framework for handling uncertainty in a large-scale programme estimating the Global Burden of Animal Diseases

**DOI:** 10.3389/fvets.2025.1459209

**Published:** 2025-03-07

**Authors:** Helen E. Clough, Gemma L. Chaters, Arie H. Havelaar, K. Marie McIntyre, Thomas L. Marsh, Ellen C. Hughes, Wudu T. Jemberu, Deborah Stacey, Joao Sucena Afonso, William Gilbert, Kassy Raymond, Jonathan Rushton

**Affiliations:** ^1^Department of Livestock and One Health, Institute of Infection, Veterinary and Ecological Sciences, University of Liverpool, Liverpool, United Kingdom; ^2^Lancaster Medical School, CHICAS, Lancaster University, Lancaster, United Kingdom; ^3^Department of Animal Sciences, Emerging Pathogens Institute, University of Florida, Gainesville, FL, United States; ^4^School of Natural and Environmental Sciences, Newcastle University, Newcastle, United Kingdom; ^5^School of Economic Sciences and Paul G. Allen School for Global Animal Health, Washington State University, Pullman, WA, United States; ^6^Department of Veterinary Epidemiology and Public Health, International Livestock Research Institute, University of Gondar, Gondar, Ethiopia; ^7^School of Computer Science, University of Guelph, Guelph, ON, Canada

**Keywords:** uncertainty, animal disease, disease burden, framework, model, estimation

## Abstract

Livestock provide nutritional and socio-economic security for marginalized populations in low and middle-income countries. Poorly-informed decisions impact livestock husbandry outcomes, leading to poverty from livestock disease, with repercussions on human health and well-being. The Global Burden of Animal Diseases (GBADs) programme is working to understand the impacts of livestock disease upon human livelihoods and livestock health and welfare. This information can then be used by policy makers operating regionally, nationally and making global decisions. The burden of animal disease crosses many scales and estimating it is a complex task, with extensive requirements for data and subsequent data synthesis. Some of the information that livestock decision-makers require is represented by quantitative estimates derived from field data and models. Model outputs contain uncertainty, arising from many sources such as data quality and availability, or the user’s understanding of models and production systems. Uncertainty in estimates needs to be recognized, accommodated, and accurately reported. This enables robust understanding of synthesized estimates, and associated uncertainty, providing rigor around values that will inform livestock management decision-making. Approaches to handling uncertainty in models and their outputs receive scant attention in animal health economics literature; indeed, uncertainty is sometimes perceived as an analytical weakness. However, knowledge of uncertainty is as important as generating point estimates. Motivated by the context of GBADs, this paper describes an analytical framework for handling uncertainty, emphasizing uncertainty management, and reporting to stakeholders and policy makers. This framework describes a hierarchy of evidence, guiding movement from worst to best-case sources of information, and suggests a stepwise approach to handling uncertainty in estimating the global burden of animal disease. The framework describes the following pillars: background preparation; models as simple as possible but no simpler; assumptions documented; data source quality ranked; commitment to moving up the evidence hierarchy; documentation and justification of modelling approaches, data, data flows and sources of modelling uncertainty; uncertainty and sensitivity analysis on model outputs; documentation and justification of approaches to handling uncertainty; an iterative, up-to-date process of modelling; accounting for accuracy of model inputs; communication of confidence in model outputs; and peer-review.

## 1 Introduction

Healthy livestock populations strengthen the health and resilience of societies, helping people to avoid hunger, access a balanced diet, generate income, and provide a pathway to local and international trade. Ill-health presents a threat to the sustainability of livestock sectors threatening livelihoods, businesses and food supply as well as wasting finite resources. Historically, there has been no systematic approach to determining which populations are at greatest risk of animal diseases. A good understanding of animal health losses can support evidence-based decision making around animal diseases and food production; it allows for better understanding of how and when these instances will occur and the development of targeted interventions for specific societies who are most severely impacted. Documenting and defining the sources of uncertainties in data used to describe livestock production systems and the diseases affecting them is central to estimating overall losses.

The Global Burden of Animal Diseases (GBADs) programme is improving the understanding of which populations are at greatest risk of animal diseases, by providing relevant metrics at both local and global levels (1). As part of its activities, the programme is capturing information describing livestock production, economic efficiency, social equity and the environment, to understand how the burden of animal health loss is disaggregated across society. Burden estimates produced by the programme are derived from a combination of field data and model outputs (2).

Alongside gathering data and parameterizing models, the GBADs programme must document sources and types of uncertainty generated through this process. Uncertainty provides substantive challenges to generating all burden estimates and knowledge describing uncertainty around these estimates should be incorporated into decisions for which they are used as evidence. The types of uncertainty in any animal production system arise from various sources. For example, understanding of animal health and production varies hugely between countries: in some localities, particularly low-income settings, there are many unknowns, and ascertaining baseline information, such as the number of animals kept, can be difficult. Additionally, data acquisition may be problematic (or impossible) in countries impacted by war, conflict and natural disaster (3). The accuracy of collected data in reflecting ‘true’ values can also be uncertain. In contrast, some countries have developed large-scale, production system-specific comprehensive animal disease surveillance programmes, following establishment of the need for such resources (4–6).

Additional uncertainty arises from how such data is analysed. Point estimates of important parameters (estimable numerical quantities) can be used to estimate disease burden, however they provide only part of the necessary understanding; the statistical, scenario-based and structural uncertainties around estimates, as well as model parameter inputs and outputs, are also important. Uncertainty should be minimized as far as possible, but where it is unavoidable it should be identified and scientifically characterized, with its sources clearly documented. Stakeholders in the programme must be confident in receiving quantitative information and knowing how to use it to make informed decisions (7). This paper presents a conversation around data and model output uncertainty against a backdrop of the work of the GBADs programme.

Statistics is the science of uncertainty in data and numbers. The predominantly statistical uncertainty discussed in this paper sits within a wider uncertainty framework (8). Uncertainty in its broadest sense has furthermore been categorized into nine types (9); epistemic, ontological and ambiguous, each of which can operate at substantive, strategic and institutional levels. Here, these types of uncertainty are used as a framework to understand how to describe uncertainty within the work of the GBADs programme. As internet and social media access has broadened, there has been greater opportunity for inaccurate information to circulate. Stakeholders, and the public, have more need than ever to understand what uncertainty means and to be able to decide what constitutes a reliable source. Fear of the unknown should be replaced with a conviction that sometimes the greatest expression of knowledge is the simple statement “this is unknown,” paving the way to address knowledge gaps.

### 1.1 Structure for the framework

The paper begins with a broad definition of models and outlines the different types of models that inform GBADs activities (conceptual; mathematical; statistical). This is followed by a description of the different types of uncertainty that exist across the GBADs analytical framework coupled with consideration of how uncertainty impacts model outputs, and a discussion of statistical principles which can be used to handle uncertainty. We include examples, based around the work of the GBADs programme, to illustrate the principles described. We conclude by describing a framework to manage and communicate uncertainty in its various forms for use by large-scale multi-partner programmes such as GBADs. Use of this framework will provide a common understanding of uncertainty and how it can be effectively handled, ensuring the rigor, repeatability, and transparency of GBADs outcomes.

## 2 The power of models in estimating the global burden of animal disease

**Figure 1:**
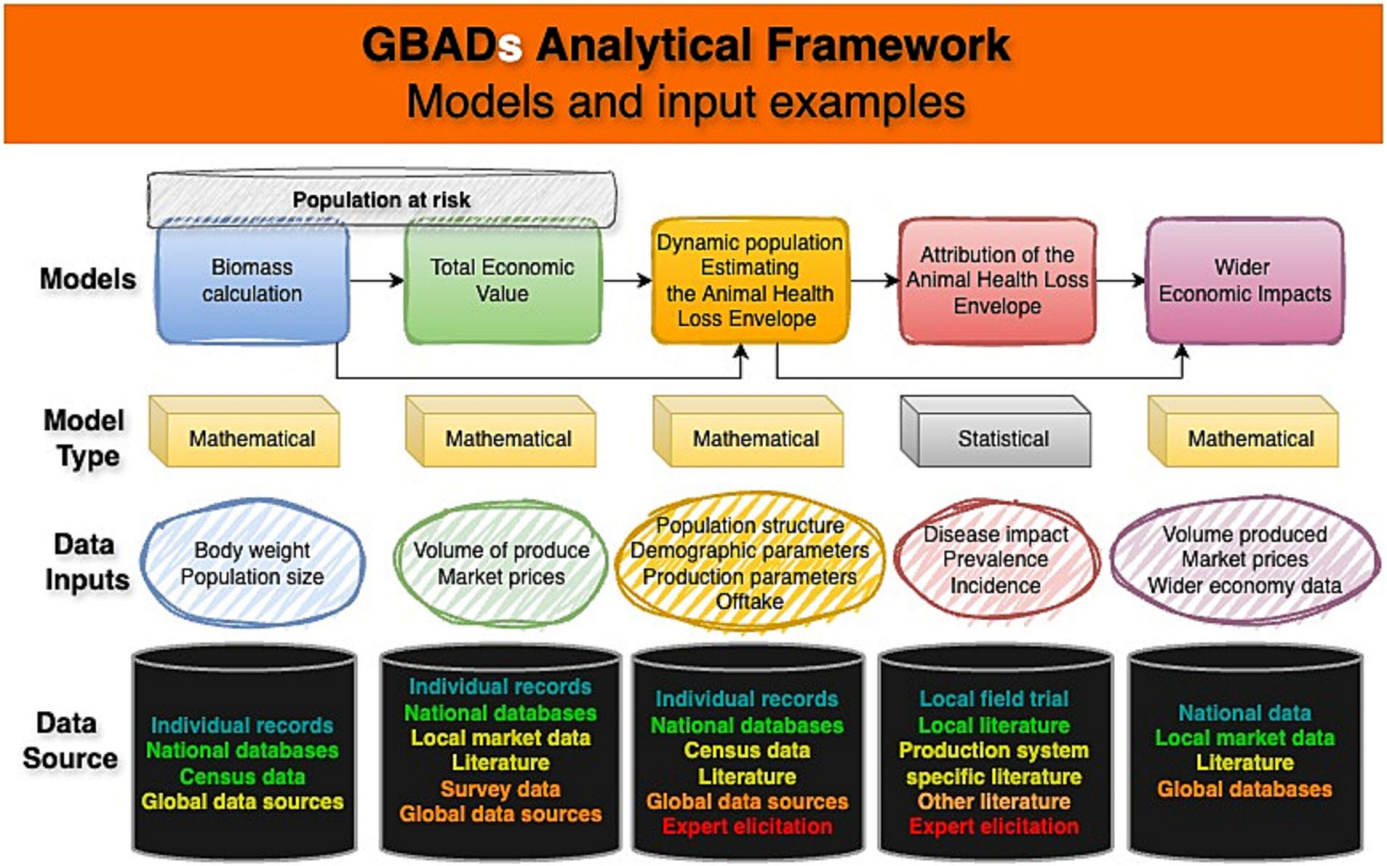
Conceptual diagram of GBADS analytical framework and data flows including example data inputs, model types and data sources, with sources color-coded (text) to indicate the evidence ‘strength’ hierarchy, with quality decreasing from turquoise-green to red. This figure adapts an established framework (1). Created using online diagram and flowchart maker draw.io.

Historically, the use of models to understand animal disease is long-established [for example, foot and mouth disease (10, 11) and bovine tuberculosis (12)]. However, approaches vary enormously. All models take inputs (parameters, informed by data or expert opinion, and assumptions) and process them in some way to produce outputs (refined information) upon which decisions may then be based. The GBADs programme is developing and using different types of statistical and mathematical population, disease and economic models within its analytical framework (13, 14). Data from different sources are synthesized and, depending on their availability, quality and traceability, used according to a hierarchy of evidence ‘strength’ (15) to minimize introduction of bias and additional uncertainty into programme outputs (Figure 1).

### 2.1 Conceptual models

**Figure 2:**
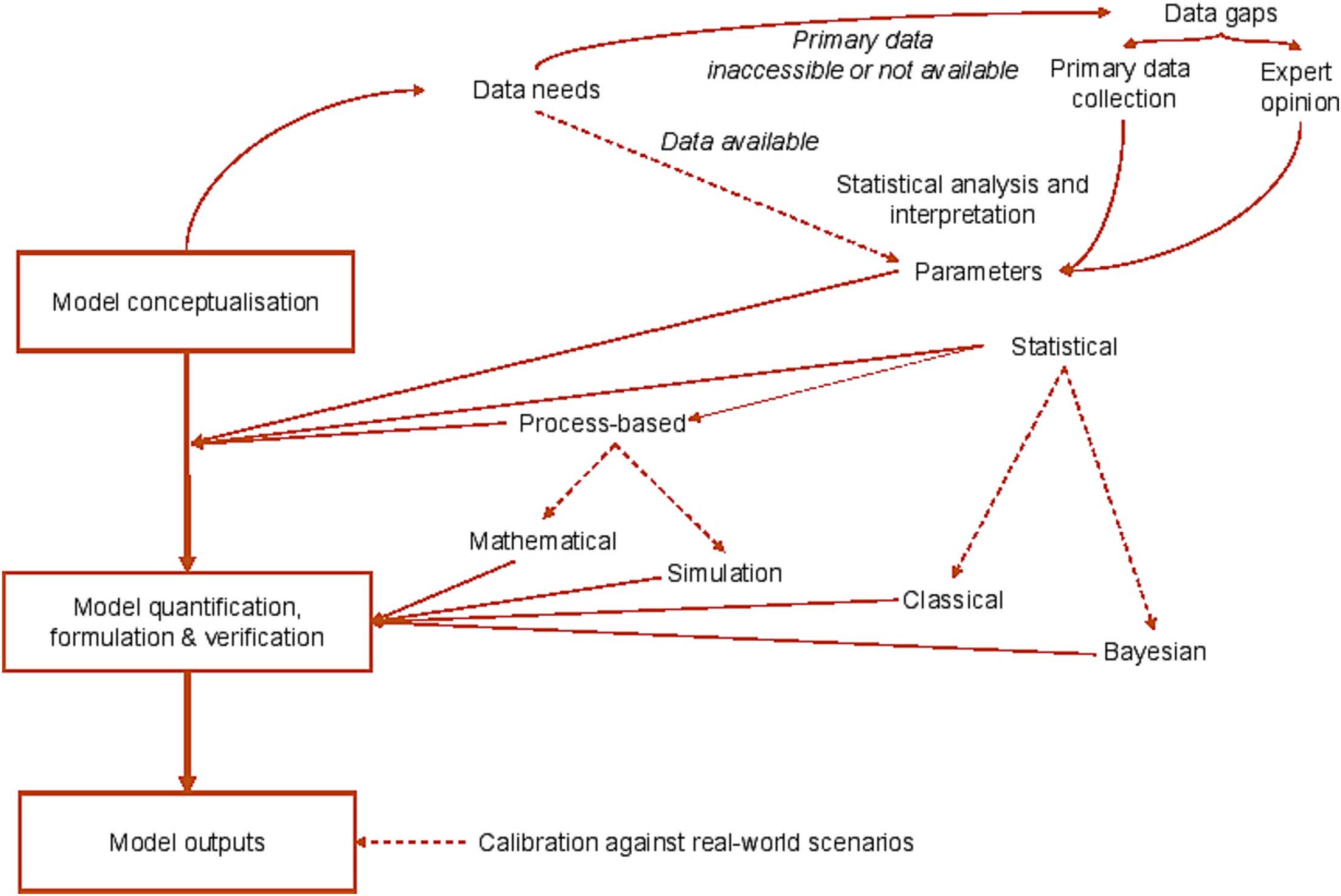
Models in GBADs: capturing model conceptualization, statistical frameworks, sources of uncertainty and information flows.

A conceptual model is often a first step in a larger modelling process and is used to describe how different parameters are linked in a system, highlight dependencies including feedback loops, and clarify required data flows (Figure 2). More formally, a conceptual model may take the form of a directed acyclic graph (DAG) (16). DAGs can help to identify confounding variables (which are linked both with the outcomes of interest and potential exposures or risk factors) (17), and are particularly helpful in large multi-factorial studies which draw upon observational data to address complicated research questions. A conceptual model may fulfil a research need in its own right, but its use is often followed by construction of a mathematical or statistical model which uses computational or simulation-based approaches.

### 2.2 Mathematical models, simulation models, and computational models

Mathematical models can provide a generalized representation of reality, for example, to facilitate the study of disease-mitigation interventions or in economics, to assess how changes in food supply will affect market prices. They are also used to simulate scenarios when there is insufficient data to build a statistical model, e.g., describing features of livestock systems and disease dynamics such as foot and mouth disease (10, 11), bovine tuberculosis (18) and the then-emergent Bovine Spongiform Encephalopathy outbreak in the 1990s (19). Mathematical models and simulation models may be deterministic (having fixed parameters, with no intrinsic randomness) or can be stochastic (including randomness) to reflect uncertainty and/or variability in input parameters.

Mathematical models incorporate critical parameters and a range of mechanistic processes, and are either represented by differential equations or, in the absence of closed-form mathematical solutions are studied via numerical methods or simulation (20–23). Mathematical models are informed by a small set of data and require prior understanding of the system being represented. Models built to describe animal production and disease dynamics (24, 25) can be subsequently modified to represent different scenarios or the impact of an intervention such as vaccination. They are also useful for the generation of hypotheses, which can then be studied in field-based investigations. Mathematical models are also used for economic assessments of animal health, including partial equilibrium models focusing on selected supply chain structures such as livestock (26), or general equilibrium models capturing sectors across the economy (13, 27).

#### 2.2.1 Examples

Suppose interest concerns the impacts of a disease on milk yield of cattle in Ethiopia but there is limited primary data. Several published articles have, however, described this disease in other settings. It is possible to build a mathematical model to describe the milk production process based on biological knowledge. Information from similar countries and understanding generated through expert elicitation (28) can then be used to inform the values of parameters in the Ethiopian system, generating hypotheses about the effects of this disease (29, 30). These mathematical models can then be used to inform the design of field studies, and data secured from these studies can be used to improve and refine the mathematical model.

### 2.3 Statistical models

Statistical modelling describes uncertainty in data sets. Statistical models are based on data and modelling assumptions, and should provide the simplest explanation for model outcomes that is not materially inconsistent with the data (31). A statistical model commonly looks for association between an outcome variable (e.g., disease occurrence) and one or more explanatory variables (e.g., environmental or behavioral factors). For the statistical model in its classical form, the sampling framework which has generated the data is important because a statistical model uses data from an appropriately-chosen sample to make inference about a population parameter.

#### 2.3.1 Examples

Statistical models have long been used to enhance understanding of disease risk factors and the effects of pathogen exposure in large epidemiological studies—for example, bovine tuberculosis (32, 33), avian influenza (34, 35), and bluetongue virus (36). Recent works have studied environmental factors associated with the spread of Highly Pathogenic Avian Influenza H5N1 virus in wild birds in Europe (37) and risks associated with African swine fever incursion in Romania (38). Using the Ethiopian dairy cattle example, a linear regression model using field data [e.g., from (39)], under appropriate random sampling-based assumptions, could be used to make inference about a relationship between cattle milk yield, livestock husbandry-associated variables, and vaccination and disease status.

### 2.4 Summary

Helpful models, conceptual, statistical and mathematical, allow researchers to understand where good data already exist, and to decide where new data are needed to improve estimates (and reduce uncertainty). The steps from conceptualization to parameterization and interpretation are therefore critical to understanding uncertainty, targeting future research priorities, and guiding how uncertainty should be managed in the future.

The types of models and the conditions under which each might be deemed suitable are summarized in Table 1.

**Table 1:** Types of models, their features and requirements.

Type of model	Data	Interest concerns process	Analytically tractable	Randomness/ variation important
Conceptual		✓		
Mathematical - deterministic	✓	✓	✓	
Mathematical - stochastic	✓	✓	✓	✓
Statistical	✓		✓	✓

## 3 Uncertainty in data and models

We begin by providing a working definition of uncertainty, set within the context of a distinction between uncertainty and risk. A long-established distinction between risk and uncertainty comes from the field of economics (40) and useful perspectives for distinguishing between risk and uncertainty have been recently explored (41). Risk relates to an unknown outcome, but the probabilistic process driving that outcome is established. In contrast, uncertainty is a state of knowledge and may arise as a result of factors such as incomplete information, complexity, randomness (variation) and unpredictability, so that both outcome and the probabilistic process driving the outcome are unknown. It is common in the mind of the non-specialist to confuse risk and uncertainty, so that when a non-specialist talks about risk, they often mean uncertainty (41). Decision-making is focused on situations where risks may be present, and decision-making in the additional presence of uncertainty is particularly challenging. In this paper we principally focus on uncertainty, rather than risk, and the different types of uncertainty are outlined below.

### 3.1 Types of uncertainty

Uncertainty occurs in all data and models; statistical, mathematical, and purely conceptual. Types of uncertainty fall into two categories (42):

1. Epistemic (knowledge) uncertainty refers to the state of knowledge and can be reduced by collecting more data. Epistemic uncertainty is intuitively well-represented by Bayesian approaches, which represent uncertainty in terms of probabilities, but we can still gain insight into epistemic uncertainty within a classical (otherwise termed frequentist) framework, for example by paying careful attention to aspects such as model selection. In practice, classical and Bayesian frameworks are both used to represent uncertainty in models, but there are distinctions between them [see, e.g., (43)]. Epistemic uncertainty can be further reduced by improving the fit of models to data. For example, suppose there are livestock growth rate estimates around which there is a large degree of uncertainty (reflected in wide confidence intervals around parameter estimates). If the population is stratified by age and breed prior to modelling, estimates may have less uncertainty around them, since growth rates within groups are likely to be more similar than those in different groups. We focus upon the acquisition of data at sub-national and production system-specific levels precisely because there is a need to reduce uncertainty in estimates reported by the GBADs programme. Further, epistemic uncertainty depends on context and will in part be influenced by the knowledge at the point of modelling; keeping estimates regularly updated can reduce epistemic uncertainty.
2. Aleatory uncertainty (sometimes termed variability) is caused by intrinsic randomness within systems. One cannot reduce aleatory uncertainty; one can only ever seek to estimate aleatory uncertainty more precisely. In agricultural systems, aleatory uncertainty can be driven by factors such as environmental variation (e.g., weather and climate) which might cause uncertainty around future production and yield, as well as price variation, which can cause market uncertainty (44). Note that aleatory uncertainty can be a feature of both statistical and stochastic mathematical models; the latter may include specific factors that cause variability, but these are underpinned by truly random processes that cannot be explained.

That many model parameters combine epistemic and aleatory uncertainty should be noted. For example, a national estimate of a parameter provides useful information, but may be of limited use at local level due to variability between sub-populations exposed to different hazards and for whom risks differ. This was demonstrated in the GBADs Ethiopian case study as stakeholders appreciated national-level estimates of disease burden but those making decisions on where to spend animal health resources requested more specific regional estimates to support their decision-making process as livestock keeping practices, pathogen challenge and resource allocation vary by region. When estimating the global burden of animal disease, intrinsic randomness and variability persist throughout the analytical framework, which makes accounting for this and describing it exceptionally important. Populations of individuals that vary are being modelled, through time, and under fluctuating environmental circumstances. Further, these populations operate within socio-cultural and political contexts that affect market supply, demand and access, impacting price variability, and with disease burdens that vary through space and time depending on the hazard (individual impacts, density and frequency dependent transmission, etc.). All these factors will contribute to final model-estimate uncertainty and must be clearly communicated to the end users of GBADs model outputs.

All models are subject to different types of uncertainty (45). Epistemic (knowledge) uncertainty can be further broken down into different categories as follows (46):

- Parameter uncertainty. This is the type of uncertainty that conventionally receives the most attention (“The best guess of the value is *x*, but it could be as low as *y*, or could be as high as *z*”).
- Model inadequacy impacts upon how well models represent reality.
- Measurement or observation uncertainty which may link to biases in collected data and in missing data. The reasons for data missingness matter.
- Residual uncertainty is “everything which cannot be explained or measured” by the model used and should be clearly reported.
- Uncertainty in the code used to represent a model.

In general, it is not possible to run a model with every possible input parameter value. Practical constraints dictate that only a subset of scenarios can be explored, in which case the uncertainty which this introduces, in terms of the range of code outputs that can be represented, must be accommodated.

### 3.2 Examples of uncertainty relevant to understanding the global burden of animal disease

#### 3.2.1 Uncertainty in mathematical models

The simplest mathematical model (47) for describing disease dynamics has three compartments: susceptible (uninfected individuals who can catch the disease when exposed); infectious (individuals who have the disease and are actively able to transmit it); and recovered (or removed), which may mean that they are immune or deceased. These models are commonly referred to as SIR models. Parameters which control the movement of individuals between these groups are commonly denoted *β*, the infectious contact rate, and *γ*, the recovery rate, and these disease-specific SIR models can be deterministic or stochastic, where elements of epistemic and/or aleatory uncertainty can be included and solvable either mathematically, or via computer simulation.

In real scenarios, mathematical models are unlikely to be so simple. Commonly, levels of complexity are included in models (age stratification; waning immunity; environmental transmission; social network-based transmission) but efforts to make models realistic may be limited by data. In mathematical models, the nature of all these mechanisms is subject to the types of uncertainty described below.

#### 3.2.2 Uncertainty in statistical models

The types of uncertainty in statistical models are well-illustrated by a cross-sectional study conducted in 13 regions of Borena zone, Ethiopia, which investigated the prevalence of Foot and Mouth Disease (FMD) in cattle (48):

- The overall within-zone prevalence of FMD in Borena zone is subject to epistemic uncertainty. Prevalence is unknown because it is not practically feasible to test every single cow in this zone for FMD. The estimated overall prevalence in cattle across Borena zone from the study was 42.7% (95%: frequentist confidence interval (CI) 37.7–47.8). Were it possible to test all animals in the zone, one could, in theory, establish the true overall prevalence of FMD in that area. Hence, the uncertainty here is a feature of the state of information (which is limited by sampling).
- The district prevalence of FMD in the zone is additionally subject to aleatory uncertainty because it varies from district to district throughout the zone. The study noted that estimates of prevalence in individual districts ranged from 25.6% (95%: CI 13.8–41.0%) to 65.5% (49.4–78.5%).

#### 3.2.3 Uncertainty arising from data and data flows

The data used in models provide the first level of uncertainty due to numerous factors including, but not limited to:

- Incomplete knowledge of how data is collected or imputed;
- Inconsistencies in collection and storage;
- Inconsistencies in availability of some types of data;
- Incomplete or missing provenance and/or metadata.

We can draw useful insight from FAOSTAT.^1^ On examination of the population numbers for animals in FAOSTAT table QCL, which provides information on production of crops and livestock products, one finds that each data point (population per species per country per year) has a flag that corresponds to Official, Estimated, Imputed, and Non-FAO. It can be difficult from associated metadata to make detailed estimations on the uncertainty to be attached to each data point. While metadata provides a guide as to how data points have been estimated or imputed, this process itself might be affected by the nature of the data collection within countries and the time at which this data point was estimated/imputed. It is important to be able to answer questions such as “does this quantity vary over time?”

It can be difficult from data sources which describe animal populations to know when numbers were collected. This is important since the point in the life cycle at which the census is taken will affect the size of the population. Some data sources, such as the Irish Agricultural Census, provide multiple population sizes with the dates at which they were collected, and variations in numbers likely reflect a combination of different types of uncertainty. Within this temporal collection framework, uncertainty is more pronounced for certain species because of the length of lifespan and the production system being used. For example, in all-in-all-out systems in which animals are bought in, fattened, and sent for slaughter (e.g., broiler chickens or intensive growing pig units), populations will fluctuate from a maximum to zero in a short space of time.

There can be inconsistencies in data sources, including large increases or decreases (>100%) in population numbers and inconsistent reporting of zero populations. For example, if a species is not present in a country should that be represented by a zero in the dataset, or an absence of reporting on that species? Clarity of meaning of zeros is important for accurate interpretation and subsequent appropriate usage of data.

The most important source of information about data is the metadata or provenance of every data point. Many agricultural datasets have limited (or no) metadata to explain how the data were collected and processed. Many groups are focusing on creating strong governance procedures to improve comparability across agricultural data sources. Without machine readable metadata using standardized metadata vocabularies (such as schema.org), the process of estimating uncertainty is hampered.

#### 3.2.4 Uncertainty arising from data access

An additional source of uncertainty arises when there are constraints around data access. The research community must work together to ensure that data can be shared safely wherever possible, and the FAIR principles (Findability, Accessibility, Interoperability, and Reusability) offer several valuable guiding points (15).

First, ‘FAIR’ principles encourage the provision of richly annotated metadata (78), reducing a source of uncertainty and allowing for the uncertainty that is present to be better understood. For instance, structure and quality of data may introduce uncertainty in models (see section 4.2.2. “Ignoring uncertainty in data”). If the structure and quality is included in the metadata, the level of uncertainty within the data can be better understood.

**Figure 3:**
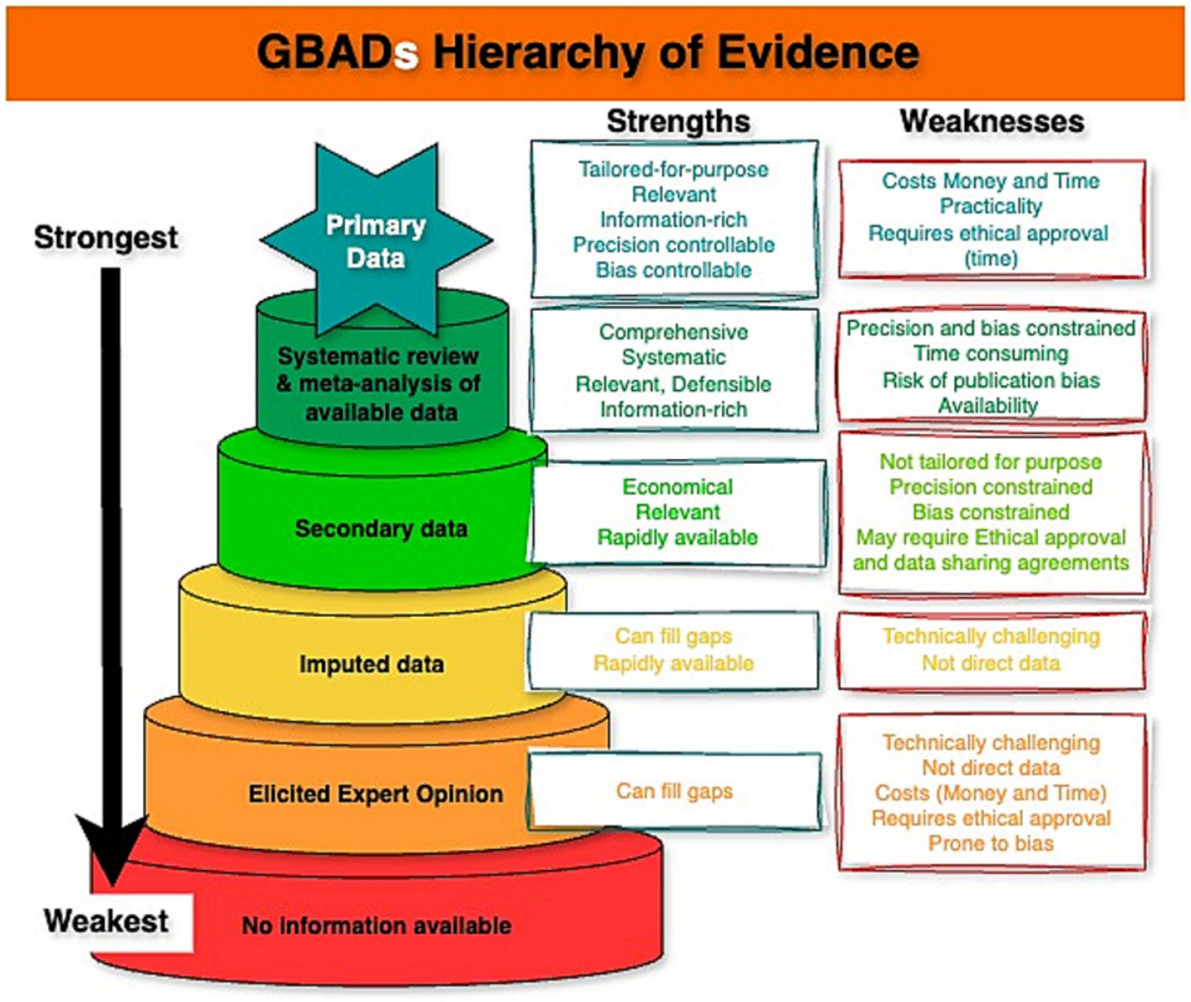
Hierarchy of Evidence used to parameterise models used to estimate the burden of animal diseases. Created using online diagram and flowchart maker draw.io.

Secondly, data types that include probability distributions (see section 4.3 “Acknowledge uncertainty, but not reflecting it properly”), can be expected to be stored, documented, and disseminated using different, yet complimentary data infrastructure, compared to raw data. Beyond the utility of metadata to ensure data are documented and uncertainty can be accessed swiftly and consistently, the framework presented in this paper describes the types of data that can be expected across models. This information is useful when conceptualizing how GBADs expects to disseminate FAIR data (e.g., “born FAIR” data) to avoid idiosyncrasies that may introduce uncertainty when new analysis reuses distributions which were primarily created for GBADs modelling. Further, as models, parameters, and outputs are updated and move up the evidence hierarchy (Figure 3), data documentation and versioning becomes important to ensure that the estimates are reproducible and can be traced with proper provenance.

While adhering to the FAIR Guiding Principles can allow practitioners to better understand the level of uncertainty in their models, reduce uncertainty in parameters and outputs, and allow others to understand the uncertainty in inputs and outputs, the livestock sector has been identified as data and metadata-poor (49) and without consistent or adequate data standards (50). A way forward is to use cases of secondary data (livestock population, weights, etc.), and to identify the information required for understanding the potential sources of uncertainty in the data. This information can take the form of additions to pre-existing metadata standards (schema.org, Dublin Core Metadata Initiative (DCMI), etc.), acting as domain-specific requirements and contributing to FAIR data. That is, rather than a user searching for documentation about data, metadata can include useful and standardized information and be provided to the user at the time of data discovery.

#### 3.2.5 Uncertainty in economic models

Dealing with uncertainty in economic modelling is achieved in several ways. First, economic models are structurally specified (firms supply and consumers demand), calibrated, and then validated. Calibration is an iterative process of comparing the model with outcomes of the economic baseline, then revising the model structure and parameters if necessary, and so on, until a model is accepted or validated (in or out of sample). Parameters in the structural equations of the model are specific to consumers and producers, meaning that uncertainty of behavior of these agents is identified and channeled through specific equations and relationships. Second, economic models which are downstream of other GBADs activities, take as input data outputs (e.g., changes in offtake or production) from modelling components within the GBADs analytical framework. If these inputs include a distribution of outcomes, then this can be simulated through the economic models to provide a distribution of economic outcomes. Subsequently, point estimates and confidence regions can be generated for relevant economic outcomes. Thirdly, sensitivity analysis can be applied to determine how changes in parameters affect the prediction of outcomes. Finally, Bayesian approaches can be applied to generate credible regions for economic outcomes, such as producer or consumer surplus (51) and to address uncertainty through model averaging (52).

### 3.3 Why does uncertainty matter?

Poor understanding of uncertainty can impact upon the ability to make good decisions. Exclusive focus on point estimates neglects uncertainty in its entirety, and this has impacts for the decision maker. On the other hand, a poorly-represented measure of uncertainty can be as damaging as no measure at all. An incorrect representation of uncertainty can either make the decision maker overly and artificially confident, or unnecessarily cautious. When uncertainty is described well the decision-maker has a full range of scenarios at their disposal. For example, suppose a study based on a random sample of one hundred herds of cattle in a region of India finds fifteen of those herds positive for an emerging disease agent. The point estimate of the herd prevalence of this new agent is then (100 × 15/100)% = 15%. The simple calculation of a Binomial confidence interval, which relates the sample-based estimate to what may be occurring in the population from which those herds were sampled, suggests that the true herd prevalence of this new agent could be anywhere between 8.7 to 23.5%, with 95% confidence.

Suppose now that the threshold for an intervention to control the disease is a prevalence of 15%, with action deemed necessary if prevalence is greater than 15%. If uncertainty is ignored, and action is based upon this point estimate alone, no intervention will take place. Alternatively, if the decision-maker understands the uncertainty, they may take a different decision since it is quite likely that the true prevalence is higher than the action level. For example, given data of 15 out of 100 positives, under certain simple statistical assumptions, the probability that the true prevalence is greater than 0.15 (meaning action would not be taken when it is needed) is 0.55.

Uncertainty also matters because levels of uncertainty may be linked with either the economic prosperity or the political and/or military stability of a country. For example, in lower income countries, which may be impacted by war, numerous factors can impact availability of and access to key sources of data and information (44), increasing uncertainty around estimates derived from such data and leading to more challenges for decision makers.

## 4 Considerations for managing uncertainty in estimating the global burden of animal disease

Uncertainty can be overlooked in a number of ways, with different difficulties arising as a result (53). Uncertainty is problematic when:

1. *A priori* objectives of a study are unclear;
2. Uncertainty is ignored;
3. Uncertainty is acknowledged, but not properly reflected;
4. Focus is placed on tangible but trivial uncertainties (rather than major, harder to quantify uncertainties);
5. Uncritical faith is placed in models and their outputs.

Points (1) to (5) (53) provide a useful framework for reflecting on and evaluating the impacts of the various types of uncertainty intrinsic to models used across the GBADs programme, where major, difficult-to-quantify uncertainties are commonplace.

### 4.1 Unclear *a priori* objectives

A first stage in any study should be to specify the research question. An intrinsic part of this involves consideration of the precise goal of the activity. If *a priori* objectives are unclear, for example, if there is a poor disease case definition, it can be difficult to establish benchmarks against which change can be measured.

### 4.2 Ignoring uncertainty

#### 4.2.1 Ignoring uncertainty in models

All mathematical models are an uncertain representation of a real-world context, and as such, mathematical models are only as good as the approximations they contain. An example from a recent GBADs Ethiopia case study (54) is use of the dynamic population model to estimate draught power production. Draught rate was only applied to castrated male animals, as this is the most common source of draught power and was supported by available data. However local knowledge of the system tells us barren female cattle are also occasionally used for draught power. This is an example of the model structure not fully representing reality and thereby introducing uncertainty, which may not be random.

In a deterministic mathematical model, every output is determined uniquely via the combination of input values. This is unlikely to be the case for most biological processes but may be a necessary step in the modelling process due to lack of data availability or resources to undertake more complex approaches. In other settings, where the assumption is that the model represents a sample of the wider population, and where data are available, a stochastic mathematical model, which allows for variation in input parameters, will be a better fit, allowing a direct reflection of at least some uncertainty. Models to estimate the global burden of animal disease incorporate stochasticity wherever data availability, quality and access allow.

As an example, animals vary in their body weight through time, but it would be too computationally expensive to model each individual, each day, to estimate population biomass. Thus in biomass and dynamic population models, individuals are grouped into age-sex strata at a level that the data allows (e.g., for Ethiopian census data cattle are 0–1 year, 1–2 years, 3 < year), and a distribution of average body weights for the mid-point of that age-sex group (95% credible interval) is sampled from 10,000 times, to produce a body weight value (biomass (kg)) that is representative of the population stratum. Uncertainty exists in the estimates here because we do not know exactly the body weight of each animal in the stratum. Conversely, when body weights are known, for example in intensive pig and poultry production, animals can be weighed through the growing process; individual animal body weights through time could be used to parameterize live weight (an example of using primary data from the top of the evidence hierarchy) in a dynamic population model, and thus these biomass model outputs would have less associated uncertainty (55).

#### 4.2.2 Ignoring uncertainty in data

The source, structure and quality of data used in models has the potential to introduce multiple layers of uncertainty. Uncertainty occurs when survey or surveillance data are treated as though they are exact, when in fact these data represent only a sub-sample of the population (56, 57). Even where all individuals in a population are accounted for, such as in a census count, the resulting number is true only at the time of the census, introducing uncertainty if the resulting value is used to represent the same population at a different time. Survey, snapshot census and surveillance data feature lower down the evidence hierarchy in comparison to datasets that monitor all individuals in a population over time. For example, in the UK and Ireland, every bovine is identified from birth and followed through its life to death using a passport and centrally recorded data (58) (strong data structure, less uncertainty). Contrasting this, in Ethiopia no similar data collection structure exists; in this setting GBADs relies on survey and census data to infer estimated livestock population size and structure (weaker data structure with greater uncertainty) (59).

Disease-reporting data, that in GBADs is used in attribution of the animal health loss envelope (AHLE) (60), must be assessed to check if it accounts for the fact that most microbiological tests are imperfect. Correcting for this can both reduce uncertainty and reduce bias; a prevalence estimate corrected for uncertain Sensitivity and Specificity will include a greater amount of uncertainty overall than the uncorrected prevalence, but will present a more realistic representation of the true state of knowledge concerning the true number of infected animals or people in the population of interest.

### 4.3 Acknowledging uncertainty, but not reflecting it properly

Sometimes, the presence of uncertainty is acknowledged, but approaches to representing uncertainty vary in their robustness. A good example of where uncertainty can be reflected uncritically is through the arbitrary selection of probability distributions, for example a triangular distribution to represent a parameter in a process-based model. A triangular distribution may at first seem an attractive option due to its intuitive parameterization in terms of its minimum, most likely and maximum values. For the incorporation of expert opinion, when it is easy for an expert to provide a guess at a minimum, most likely and maximum value for a parameter, or for survey data from relatively small samples, this distribution can be favored. However, this distribution is likely a flawed representation of most biological processes as the triangular distribution is fixed as linear between the minimum, mode and maximum with artificially strict lower and upper bounds and heavier tails than is likely to be realistic (61). Limitations of such distributions must be acknowledged, and it is important that GBADs models, parameters and outputs are regularly updated, moving up the evidence hierarchy, when more complete data become available, through a transparent dataflow processes. A modification of the triangular distribution is a Pert distribution, which is less heavy in its tails and has been used to parameterize sections of the dynamic population models in the GBADs analytical framework applied in Ethiopia, Senegal and Indonesia [for example (62)].

### 4.4 Tangible but trivial uncertainties

Tangible but trivial uncertainties are uncertainties to which people can relate but which have very minor impact should they occur. Examples include uncertainty around the probability that one cattle feed will produce a greater milk yield than another cattle feed; this is a parameter to which people can relate, but the choice of one feed or another is likely to have minimal impact upon milk yield, if both have been shown in previous studies to perform well. Contrast this with uncertainties which may or may not be tangible, but are important – for example, the probability of a catastrophic drought in the next 12 months (tangible, in that people can conceive what it means, and important, because the impacts it may have are clear); or the probability of an as-yet unseen infectious disease emerging in the next decade (less tangible, an example of an “unknown unknown,” and important, because it has the potential to make ill or even destroy animals and/or humans, and will need to be managed when it occurs). Sensitivity analysis (63), can be a useful body of methods to understand where these tangible but trivial inputs are, and what their effects on overall output uncertainty might be.

### 4.5 Uncritical faith in models and outputs

No model outputs should be communicated as “the truth,” only as a fair and/or likely representation of reality with the associated uncertainties clearly described (64). For example, a mathematical model for one disease applied directly to represent another has the capacity to mislead if the underlying population structure, disease dynamics and interactions within the population differ. In any attempt to apply a model from one context to another, areas of uncertainty and key assumptions must be documented and this is a critical stage in the framework for handling of uncertainty in estimates contributing to the assessment of the global burden of animal disease. As data availability increases, GBADs models will continue to be refined to represent production system specific scenarios and locally relevant, population and pathogen dynamics and economies.

Model uncertainty and the impact of different model assumptions can be explored in a number of ways. Model averaging, as described in (65), is an approach to estimation which accommodates model uncertainty by taking a weighted average of estimates from candidate models, where weights in some way reflect how plausible the model is for the scenario under study. Alternatively, fully Bayesian approaches have been proposed (45) which can be tailored to calibrate model outputs against known data sources. Note that these approaches require a high-level understanding of statistics and are best approached with specialist statistical input.

## 5 Towards an uncertainty framework for estimating the global burden of animal disease

In the following section we summarize recommendations for dealing with uncertainty to strengthen the value and reliability of outputs from the large-scale GBADs programme, which draws extensively upon models of multiple types. Figure 2 summarizes the role of models in the programme across the analytical framework.

**Figure 4:**
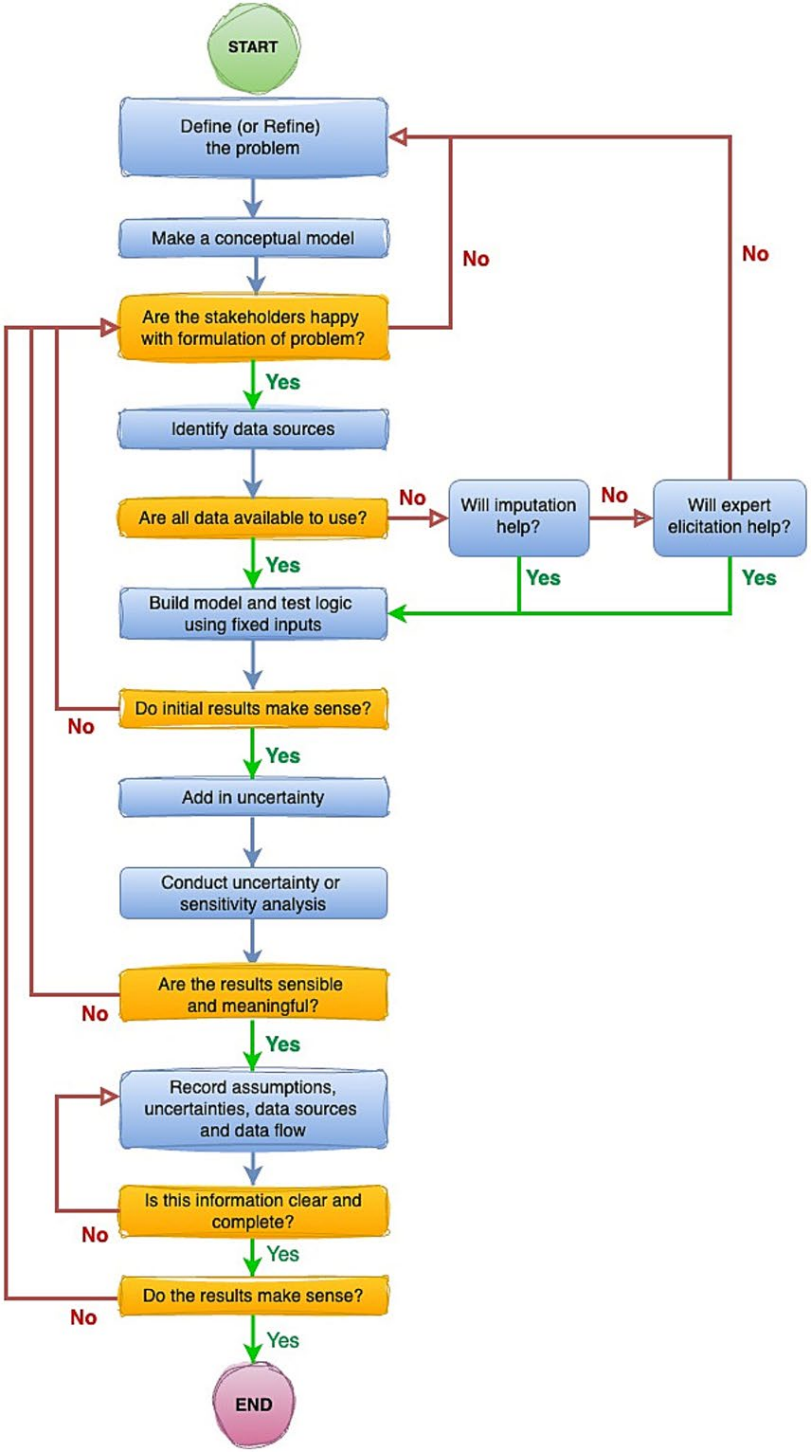
Stepwise pathway to ensure appropriate considerations of uncertainty are embedded at all stages of estimation of the global burden of animal diseases.

**Figure 5:**
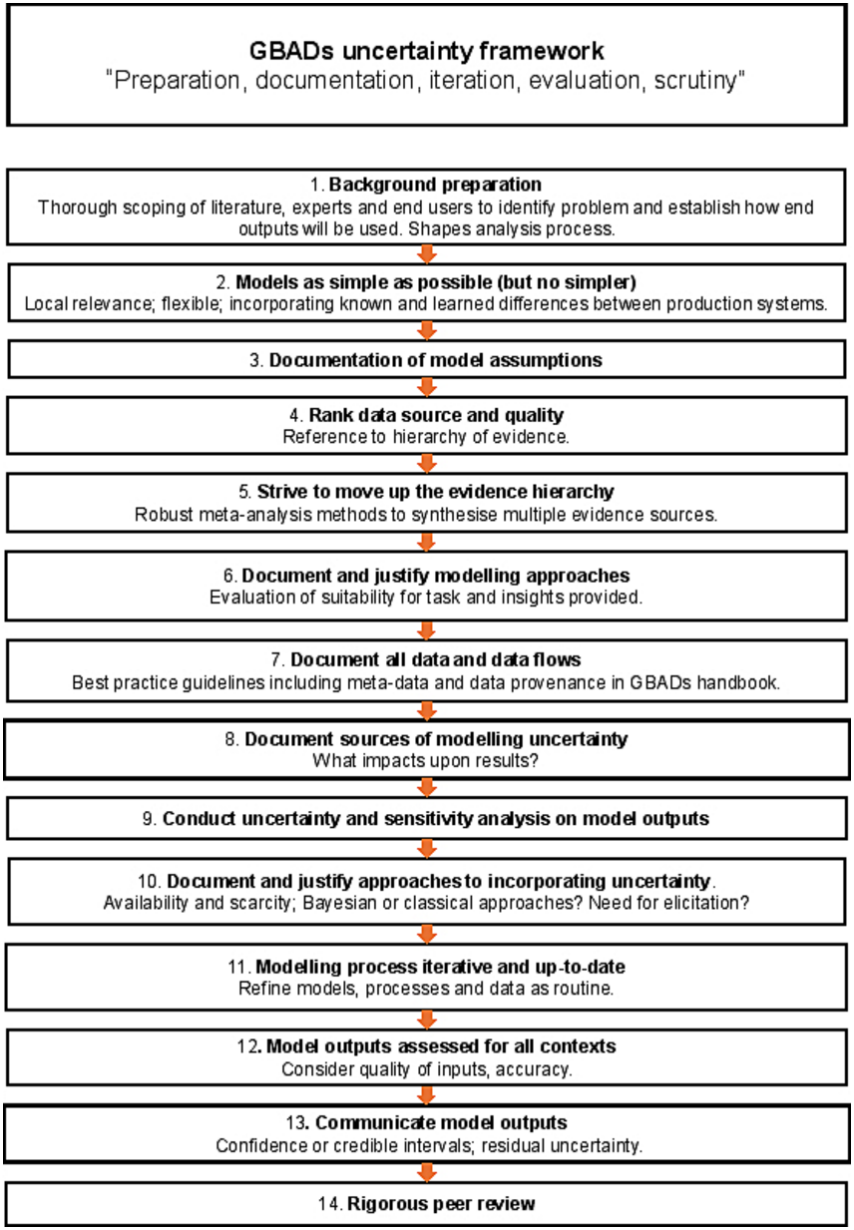
A generalized framework for uncertainty in estimating the global burden of animal disease.

Important authors in the field of uncertainty define a set of “ten commandments for good policy analysis” (66). We use these and ideas of others around management of uncertainty (67, 68), together with our own reflections and experience within the programme so far (69), to,

1. Outline a hierarchy of evidence (Figure 3) which is intended to guide movement (Figure 4) from worst through to best-case sources of information to inform modelling, and
2. Suggest a suitable framework setting out an overall position for handling uncertainty in estimating the global burden of animal disease (Figure 5).

The means by which GBADs deals with uncertainty and supports users of our information in its interpretation for decision making on animal health are many-fold. For an “optimal” approach to uncertainty in a programme of this nature, we suggest that the following steps (visualized in Figure 5) should be followed:

1. Background preparation should involve thorough scoping of the literature, the relevant body of experts, and end-users of the global animal disease burden that constitutes the principal GBADs output. The specific problem being addressed should shape the chosen analysis (66), commandments 1 and 2.
2. The analysis should be as simple as possible, but, no simpler (66), commandment 3—a parsimonious model. A simple model can be easier to explain and for people to understand, and as a bonus generates less parameter uncertainty; but an analysis that is over-simplified risks missing important features (for example, neglect of a particular disease transmission route or production process).
3. All assumptions feeding into the modelling process must be clearly stated (66), commandment 4, and there must be a clear understanding of how the outputs are going to be used, and what the criteria for making decisions are going to be (for example, if more than 5% of animal health losses are due to disease X, routine vaccination will be implemented), commandment 5.
4. Multiple types of evidence must be brought together across the GBADs analytical framework to provide a comprehensive overview of the costs of animal health losses (1). The quality of all data should be ranked according to an evidence hierarchy; we suggest the hierarchy in Figure 3.
5. There should be an ongoing process amongst all partners of striving to move up the evidence hierarchy whenever possible. Models and estimates should be kept current and data used for parameterization should be refreshed at each opportunity as better sources or methods become available, allowing the analytical process to climb up the hierarchies of evidence ladder in a timely and systematic way.
6. Proposed approaches to any necessary modelling should be documented and justified. Mathematical, statistical and simulation models are all within scope; in each case, the approach should be justified in terms of the insights it will bring and its suitability for the task.
7. Data sources should be documented and described in associated metadata and data flows as completely as possible. Any barriers to data access and data sharing should be identified early in the process, alongside any stipulations around where and how data should be hosted, requirements around data security and governance. Transparency in the use of data and documentation is paramount.
8. All sources of uncertainty should be described and documented as early as possible in the process (66), commandment 6. This should include consideration of qualitative uncertainties, for example organizational and political uncertainty, alongside data-associated considerations. This should be followed by discussion around which uncertainties matter and which, *a priori*, are believed to have most impact upon results.
9. Thorough sensitivity and uncertainty analysis should be undertaken as part of this process (66), commandment 7 and should inform priorities in data collection.
10. Proposed approaches to the incorporation of uncertainty in each of the selected models should be documented and justified. Bayesian and classical statistical approaches should be a first choice when useable data have been identified and sourced; expert opinion-based approaches may be used (see 4 and 5); alternately, a more arbitrary choice of probability distributions to describe uncertainty may be used but choice of both technique and distribution should be clearly argued and documented, along with a consideration of any weaknesses or biases introduced by the chosen approaches.
11. All parts of the process should be iterative, so that the statement of the problem is refined should it be needed, and the analysis methods refined accordingly (66), commandment 8. All parts of the process should be supported by clear and complete documentation (66), commandment 9.
12. For a given context, there should be a final assessment of overall quality of outputs, which accommodates the accuracy and quality of inputs from the previous stages.
13. Residual uncertainty should always be documented and described as fully as possible. It is important that all stakeholders are clear about what a model can and cannot explain. Confidence intervals or credible intervals describing uncertainty should be reported rather than *p*-values whenever possible. To justify this, it is helpful to reflect upon the fact that a *p*-value in isolation, without associated reporting of uncertainty, is of little value to the decision maker, but a confidence interval provides a range (68), that is, an estimate of best- and worst-case scenarios (“the effect could be as small as this, or as big as this”) which can be very useful when scrutinized against the appropriate background context for informing decisions.
14. All outputs should be subjected to a process of peer review (66), commandment 10.

The framework presented in Figure 5 draws upon a similar framework (66) which refines a stepwise process to ensure that clear scrutiny of what is known well, what is uncertain, and what is completely unknown, is embedded in decision-making processes. In the spirit of reporting uncertainty in full at all stages, it is not sufficient to multiply point estimates together to produce a model output. Point estimates for key model parameters should be reported, carefully, but it is equally important that any “best guesses” are accompanied by measures of uncertainty, represented by probability distributions where possible.

### 5.1 GBADs example

A recent case study attributing Ethiopian animal health losses to high-level causes can be used as an exemplar of the GBADs uncertainty framework in action (69). The paper describes a method for undertaking an expert elicitation to obtain the data needed to attribute an Animal Health Loss Envelope AHLE—a novel GBADs metric (60, 70) to infectious, non-infectious and external causes. The AHLE is calculated using a dynamic population model to simulate herd growth and expenditure.

- First, the problem was defined and a thorough background scoping review undertaken, including identification of attributable causes of animal heath loss, exploration of possible data sources (both primary data and literature), flagging of the likelihood of data gaps, and exploration of possible methods for filling those data gaps, including evaluation of the appropriateness of different methods for structured expert elicitation (*step 1*).
- Although no complex models were needed for this study, data collection was structured to provide the most information in the simplest form, important to ensure adherence to step 2 of the GBADs uncertainty framework and also to ensure clarity of understanding by experts. Experts were asked for their minimum, maximum and most likely estimates for each type of high-level cause. A beta-Pert distribution was chosen to model these estimates over, for example, a simpler but rather more crude, triangular distribution, which incorporates bias and can be overly confident in the distributional tails (*step 2*).
- The inherent assumptions that accompany the selected approaches were documented and discussed (*step 3*). The data were collected and analysed in the context of a well-defined downstream aim (i.e., high-level attribution of an AHLE in Ethiopian livestock).
- Data were derived from a single source, structured expert elicitation, and so ranking of the data source was not required (*step 4*).
- The limitations of expert opinion are acknowledged in the study and the intrinsic superiority of richly data-based methods drawing upon a variety of sources that, at the time of writing, were in preparation is recognized (*step 5*). Although elicited expert opinion is low down on the (GBADs) hierarchy of evidence (Figure 3) steps were taken to mitigate this: first, in step 1, other potential sources of data for this study were explored and were found to be unavailable at the time of the study; secondly, a structured, transparent and well-documented method (the IDEA protocol), which has been subjected to peer review in previous studies (79), was used to collect the data from recognized experts in the local domain of interest. This enabled biases in the data to be recognized and minimized.
- The paper documents and justifies all analytical approaches (*step 6*) via a thorough description of the structured elicitation protocol and choice of probability distributions (models).
- The methods of data collection, processing (including anonymization) and analysis are described. Raw data is made available in the supplementary materials (*step 7*) of the manuscript (69).
- Uncertainty receives primary consideration throughout the study and was integral to the development of the study design (*step 8*). For example, qualitative uncertainties are acknowledged from the outset in that different experts will have different opinions. The IDEA protocol was used to account for this, through discussion between experts and statistical aggregation of individual opinions. Additional quantitative sources of uncertainty were acknowledged and accounted for through the choice of distributions.
- Critical uncertainty analysis was undertaken and approaches to tackling uncertainty informed and was informed by how data was collected (see above). Consideration of the impacts of uncertainty on the estimation of high-level attribution is explained (*step 9*) and clear documentation of the approach used to represent uncertainty is included (*step 10*).
- In all GBADs activities there is a commitment to ongoing refinement of models as new information sources become available (*step 11*). This study represents an initial estimation of high-level attribution of animal health losses but work is ongoing, both the move up the data hierarchy by developing data-driven attribution methods (13, 70) and where expert elicitation approaches are needed to refine and improve these methods to minimize and better capture uncertainty.
- Critical assessment of the results of this study, both in term of the expert’s estimates and their implications for attribution of health losses to external, non-infectious and infectious causes, was undertaken and discussed at length (*step 12*).
- Throughout, all results are communicated in terms of uncertainty intervals (*step 13*) and finally, the whole exercise was subjected to peer review resulting in its publication in an internationally-recognized journal (*step 14*).

## 6 Discussion

Complex analytical frameworks that involve running multiple types of model, using data from a range of sources (from national statistical databases to expert elicitation), have provided the foundations for the work undertaken to estimate the Global Burden of Diseases in human populations, which has helped to focus investment in human health at a global scale (71, 72). The Global Burden of Animal Diseases (GBADs) programme is now working towards improving investments in animal health using similar analytical approaches, employing a variety of models, and even more varied sources of data given the de-centralised nature of animal health information. As outlined in this paper, uncertainty, around data inputs and outputs, as well the models themselves, is an unavoidable aspect of such analyses, but identifying sources of uncertainty and taking steps to account for and reduce them, are vital to producing estimates of animal disease burdens that can best inform decision makers.

Decision makers who use models to inform their decisions should never assume that their models equate to reality; the eminent statistician George Box famously said “all models are wrong, some are useful.” A key acknowledgement should be that the outputs from a model do not provide a correct representation of the real world. Nevertheless, when used well, model outputs can improve the decision-making process when compared to using raw data alone. Instead of relying on model outputs, the scientist or the decision maker must always ask:

- Given that I cannot assume this model is correct, is this model useful?
- If so, how?

A key part of being able to judge whether a model is useful is making sure that uncertainty at all levels and of all types is recognized, documented, and approaches to handling that uncertainty are transparent. This paper sets out a framework which can help to ensure that this end is achieved, and that uncertainty in its various forms means the same thing to all partners in a large and complex programme.

There are many types of uncertainty. Our focus here has been on the impacts of quantitative uncertainty as is evidenced in mathematical and statistical models and data, but there can be additional uncertainty around the behavior of individuals, organizations, policy makers and around, for example, the impacts of factors such as political instability. Given this, a trans-disciplinary programme such as GBADs is ideally equipped to establish a common language for uncertainty, and to take account of uncertainty in its multiple forms – in terms of designing models and important processes to be taken into consideration, as well as more obvious quantitative considerations. All of this moves to an approach to accommodating consideration of “total uncertainty” in a large and complex programme.

A formal framework for following the modelling process and ensuring common approaches to uncertainty throughout is important because it ensures repeatability of the analysis and guarantees transparency of approach and assumptions. It is important that any future user, who is equipped with a description of the chosen approaches and data sources, would be able to replicate the findings.

There is a clear need to separate the quality of the evidence from the strength and consequence of the recommendations, and from the consequences of the decisions made using model outputs. Evidence quality might be graded in qualitative terms (“weak,” “average,” “strong” evidence) or where possible quantitative terms (probability that the evidence is reliable, perhaps accompanied by an assessment of the degree of confidence in the estimated probability) (see, for example, (73)). Consequence of the recommendations again might be expressed qualitatively (“no consequence,” “mild consequences,” “severe consequences,” with positive and negative angles both being possible). Consequence of decisions might then involve exploring a range of “what if” scenarios, which could themselves be studied using risk assessment frameworks, which can either be parameterized in qualitative or quantitative terms.

The quality of inference for models of animal diseases can be impacted by a number of factors, for example a system in which funding for the study of animal diseases is low in comparison with funding for human diseases can result in a paucity of data (74) and this brings its own challenges. This is the reality for much of GBADs work in the animal health domain, particularly in low and middle-income countries, so strengthening disease surveillance and livestock production data collection structures is key to reducing uncertainty in burden estimates.

In terms of parting conclusions and suggestions, a key writer in this field (75) argues that, in line with many pastoral societies, we should look to embrace uncertainty as an unavoidable feature of life, rather than looking to eliminate it; the authors argue that this approach places society in the best position to respond to unanticipated events when they happen. Their working paper is clear to draw the fundamental distinction between risk and uncertainty, and a strength of our proposed framework is that it does the same. In Scoones’ work there is an interesting discussion around the fact that many disease control efforts, frequently politically underpinned, centre around early warning systems which are framed in terms of the language of risk and emergency and which are, by design, pathogen-driven. Diverse cultures can provide useful information about effective disease control strategies for their communities, which are based upon cultural practices and may draw upon input from senior respected figures. Examples are documented in human health, for example, the role of religious leaders in messaging around HIV prevention in Uganda (76) and building trust around COVID-19 vaccines in vulnerable communities in Ethiopia (77). For uncertainty to be reduced as far as possible and its effects mitigated, the widest perspective on what uncertainty means is important; this incorporates societal, cultural and political alongside scientific perspectives. All perspectives, especially representing those on the margins, have an important role to play in the handling of uncertainty.

## Data Availability

The original contributions presented in the study are included in the article/supplementary material, further inquiries can be directed to the corresponding author.

## Footnotes

^1^ https://www.fao.org/faostat/en/#home

## References

[1] Rushton J Bruce M Bellet C Torgerson P Shaw APM Marsh TL . Initiation of global burden of animal diseases Programme. Lancet. (2018) 392:538–40. doi: 10.1016/S0140-6736(18)31472-7, PMID: 30152376

[2] Huntington B Bernardo TM Bondad-Reantaso M Bruce M Devleesschauwer B Gilbert W . Global burden of animal diseases: a novel approach to understanding and managing disease in livestock and aquaculture. Rev Sci Tech. (2021) 40:567–84. doi: 10.20506/rst.40.2.3246, PMID: 34542092

[3] Idris I. Livestock and conflict in South Sudan in K4D Helpdesk Report. The Institute of Development Studies and Partner Organisations. (2018).

[4] Chaters GL Johnson PCD Cleaveland S Crispell J de Glanville WA Doherty T . Analysing livestock network data for infectious disease control: an argument for routine data collection in emerging economies. Philos Trans R Soc Lond Ser B Biol Sci. (2019) 374:20180264. doi: 10.1098/rstb.2018.0264, PMID: 31104601 PMC6558568

[5] Churakov M Katholm J Rogers S Kao RR Zadoks RN. Assessing potential routes of *Streptococcus agalactiae* transmission between dairy herds using national surveillance, animal movement data and molecular typing. Prev Vet Med. (2021) 197:105501. doi: 10.1016/j.prevetmed.2021.105501, PMID: 34624567

[6] Kao RR Danon L Green DM Kiss IZ. Demographic structure and pathogen dynamics on the network of livestock movements in Great Britain. Proc Biol Sci. (2006) 273:1999–2007. doi: 10.1098/rspb.2006.3505 16846906 PMC1635475

[7] Gigerenzer G. Reckoning with risk: Learning to live with uncertainty. penguin. (2003).

[8] EFSA. Guidance on uncertainty analysis in scientific assessments. EFSA J. (2018) 16:e05123. doi: 10.2903/j.efsa.2018.5123 32625671 PMC7009727

[9] Dewulf A Biesbroek R. Nine lives of uncertainty in decision-making: strategies for dealing with uncertainty in environmental governance. Polic Soc. (2018) 37:441–58. doi: 10.1080/14494035.2018.1504484

[10] Kao RR. The role of mathematical modelling in the control of the 2001 FMD epidemic in the UK. Trends Microbiol. (2002) 10:279–86. doi: 10.1016/S0966-842X(02)02371-5, PMID: 12088664

[11] Keeling MJ. Models of foot-and-mouth disease. Proc Biol Sci. (2005) 272:1195–202. doi: 10.1098/rspb.2004.3046 16024382 PMC1564112

[12] Brooks-Pollock E Roberts GO Keeling MJ. A dynamic model of bovine tuberculosis spread and control in Great Britain. Nature. (2014) 511:228–31. doi: 10.1038/nature13529, PMID: 25008532

[13] Hughes E Gilbert W Rushton J Bruce M Larkins A Jemberu Temesgen W . Global Burden of Animal Diseases (GBADs), Technical Guide (version 1.0) 2024. Global Burden of Animal Diseases programme. (2024). doi: 10.5281/zenodo.11093239

[14] Rushton J Huntington B Gilbert W Herrero M Torgerson PR Shaw APM . Roll-out of the global burden of animal diseases programme. Lancet. (2021) 397:1045–6. doi: 10.1016/S0140-6736(21)00189-6, PMID: 33549170

[15] McIntyre K. M. Amenu K. Chaters G. Di Bari C. Gilbert W. Jeannin M. . (2022). “FAIR evidence assessment and variable collation for the global burden of animal diseases programme: development of a futureproofed pipeline process.” In International Symposia on veterinary epidemiology and economics (ISVEE 16). Halifax, Canada.

[16] Piccininni M Konigorski S Rohmann JL Kurth T. Directed acyclic graphs and causal thinking in clinical risk prediction modeling. BMC Med Res Methodol. (2020) 20:179. doi: 10.1186/s12874-020-01058-z, PMID: 32615926 PMC7331263

[17] Tennant PWG Murray EJ Arnold KF Berrie L Fox MP Gadd SC . Use of directed acyclic graphs (DAGs) to identify confounders in applied health research: review and recommendations. Int J Epidemiol. (2020) 50:620–32. doi: 10.1093/ije/dyaa213 PMC8128477 33330936

[18] Cox DR Donnelly CA Bourne FJ Gettinby G McInerney JP Morrison WI . Simple model for tuberculosis in cattle and badgers. Proc Natl Acad Sci USA. (2005) 102:17588–93. doi: 10.1073/pnas.0509003102, PMID: 16306260 PMC1292989

[19] Ferguson NM Donnelly CA Woolhouse MEJ Anderson RM. ‘The epidemiology of BSE in cattle herds in Great Britain. II. Model construction and analysis of transmission dynamics’, *philosophical transactions of the Royal Society of London*. Series B Biol Sci. (1997) 352:803–38. 10.1098/rstb.1997.0063 PMC1691968 9279898

[20] Berriman AD Clancy D Clough HE Christley RM. Semi-stochastic models for Salmonella infection within finishing pig units in the UK. Math Biosci. (2013) 245:148–56. doi: 10.1016/j.mbs.2013.06.004, PMID: 23796599 PMC3791402

[21] Turner J Begon M Bowers RG French NP. A model appropriate to the transmission of a human food-borne pathogen in a multigroup managed herd. Prev Vet Med. (2003) 57:175–98. doi: 10.1016/S0167-5877(03)00006-0, PMID: 12609464

[22] Yang N Wu C McMillan IAN. New mathematical model of poultry egg production. Poult Sci. (1989) 68:476–81. doi: 10.3382/ps.0680476

[23] Zadoks RN Allore HG Hagenaars TJ Barkema HW Schukken YH. A mathematical model of *Staphylococcus aureus* control in dairy herds. Epidemiol Infect. (2002) 129:397–416. doi: 10.1017/S0950268802007483, PMID: 12403116 PMC2869899

[24] Dumas A Dijkstra J France J. Mathematical modelling in animal nutrition: a centenary review. J Agric Sci. (2008) 146:123–42. doi: 10.1017/S0021859608007703

[25] Tedeschi LO Menendez HM. Chapter 25 - mathematical modeling in animal production In: Bazer FW Cliff Lamb G Guoyao W, editors. Animal Agriculture. London: Academic Press (2020)

[26] Zhao Z Wahl TI Marsh TL. Invasive species management: foot-and-mouth disease in the U.S. beef industry. Agric Resour Econ Rev. (2006) 35:98–115. doi: 10.1017/S106828050001008X

[27] Corong EL Hertel TW McDougall R Tsigas ME van der Mensbrugghe D. The standard GTAP model, version 7. J Global Econ Anal. (2017) 2:1–119. doi: 10.21642/JGEA.020101AF

[28] Colson A Cooke R. Expert elicitation: using the classical model to validate experts’ judgments,” review of environmental economics and policy. Rev Environ Econ Policy. (2018) 12:113–32. doi: 10.1093/reep/rex022

[29] Lyons NA Alexander N Stärk KDC Dulu TD Sumption KJ James AD . Impact of foot-and-mouth disease on milk production on a large-scale dairy farm in Kenya. Prev Vet Med. (2015) 120:177–86. doi: 10.1016/j.prevetmed.2015.04.004, PMID: 25912977

[30] Rasmussen P Shaw APM Muñoz V Bruce M Torgerson PR. Estimating the burden of multiple endemic diseases and health conditions using Bayes’ theorem: a conditional probability model applied to UK dairy cattle. Prev Vet Med. (2022) 203:105617. doi: 10.1016/j.prevetmed.2022.105617, PMID: 35358837 PMC9127345

[31] Diggle PJ Chetwynd AG. Statistics and scientific method: An introduction for students and researchers. New York: Oxford University Press (2011).

[32] Brunton LA Alexander N Wint W Ashton A Broughan JM. Using geographically weighted regression to explore the spatially heterogeneous spread of bovine tuberculosis in England and Wales. Stoch Env Res Risk A. (2017) 31:339–52. doi: 10.1007/s00477-016-1320-9

[33] Byrne AW Barrett D Breslin P Madden JM Keeffe J Ryan E. Bovine tuberculosis (*Mycobacterium bovis*) outbreak duration in cattle herds in Ireland: a retrospective observational study. Pathogens. (2020) 9:815. doi: 10.3390/pathogens9100815, PMID: 33027882 PMC7650827

[34] Gierak A Śmietanka K. The impact of selected risk factors on the occurrence of highly pathogenic avian influenza in commercial poultry flocks in Poland. J Vet Res. (2021) 65:45–52. doi: 10.2478/jvetres-2021-0013, PMID: 33817394 PMC8009582

[35] Kuo HI Lu CL Tseng WC Li HA. A spatiotemporal statistical model of the risk factors of human cases of H5N1 avian influenza in south-east Asian countries and China. Public Health. (2009) 123:188–93. doi: 10.1016/j.puhe.2008.10.012, PMID: 19144364

[36] Ward MP. Climatic factors associated with the infection of herds of cattle with bluetongue viruses. Vet Res Commun. (1996) 20:273–83. doi: 10.1007/BF00366925, PMID: 8739526

[37] Si Y Wang T Skidmore AK de Boer WF Li L Prins HHT. Environmental factors influencing the spread of the highly pathogenic avian influenza H5N1 virus in wild birds in Europe. Ecol Soc. (2010) 15:326. doi: 10.5751/ES-03622-150326

[38] Boklund A Dhollander S Chesnoiu Vasile T Abrahantes JC Bøtner A Gogin A . Risk factors for African swine fever incursion in Romanian domestic farms during 2019. Sci Rep. (2020) 10:10215. doi: 10.1038/s41598-020-66381-3, PMID: 32576841 PMC7311386

[39] Tadesse B Tesfahun A Molla W Demisse E Jemberu WT. Foot and mouth disease outbreak investigation and estimation of its economic impact in selected districts in Northwest Ethiopia. Vet Med Sci. (2020) 6:122–32. doi: 10.1002/vms3.208, PMID: 31710180 PMC7036304

[40] Knight FH. Risk, uncertainty and profit. New York: Sentry Press (1921).

[41] De Groot K Thurik R. Disentangling risk and uncertainty: when risk-taking measures are not about risk. Front Psychol. (2018) 9:2194. doi: 10.3389/fpsyg.2018.02194, PMID: 30498464 PMC6249320

[42] Oberkampf WL Helton JC Joslyn CA Wojtkiewicz SF Ferson S. Challenge problems: uncertainty in system response given uncertain parameters. Reliab Eng Syst Safety. (2004) 85:11–9. doi: 10.1016/j.ress.2004.03.002

[43] Congdon P. Bayesian statistical modelling. Chichester, West Sussex: Wiley (2006).

[44] Rushton J. *The economics of animal health and production* (Cabi). CABI books. (2009).

[45] Kennedy MC O’Hagan A. Bayesian calibration of computer models. J R Statis Soc Series B. (2001) 63:425–64. doi: 10.1111/1467-9868.00294

[46] Mullins J Ling Y Mahadevan S Sun L Strachan A. Separation of aleatory and epistemic uncertainty in probabilistic model validation. Reliab Eng Syst Safety. (2016) 147:49–59. doi: 10.1016/j.ress.2015.10.003

[47] Kermack WO McKendrick AG. A contribution to the mathematical theory of epidemics. Proc R Soc Series A. (1927) 115:700–21.

[48] Tesfaye A Mesfin Sehale M Ashebir Abebe A Muluneh A Gizaw D. Sero-prevalence of foot and mouth disease in cattle in Borena zone. Ethiopian Vet J. (2016) 20:55–66. doi: 10.4314/evj.v20i1.4

[49] Kebede EA Ali HA Clavelle T Froehlich HE Gephart JA Hartman S . Assessing and addressing the global state of food production data scarcity. Nat Rev Earth Environ. (2024) 5:295–311. doi: 10.1038/s43017-024-00516-2

[50] Bahlo C Dahlhaus P. Livestock data – is it there and is it FAIR? A systematic review of livestock farming datasets in Australia. Comput Electron Agric. (2021) 188:106365. doi: 10.1016/j.compag.2021.106365

[51] Davis GC Espinoza MC. A unified approach to sensitivity analysis in equilibrium displacement models. Am J Agric Econ. (1998) 80:868–79. doi: 10.2307/1244070

[52] Steel MFJ. Model averaging and its use in economics. J Econ Lit. (2020) 58:644–719. doi: 10.1257/jel.20191385

[53] Milner-Gulland EJ Shea K. Embracing uncertainty in applied ecology. J Appl Ecol. (2017) 54:2063–8. doi: 10.1111/1365-2664.12887, PMID: 29225369 PMC5722456

[54] Jemberu WT Chaters G Asfaw W Asteraye GB Amenu K Huntington B . Application of global burden of animal diseases methods at country level: experiences of the Ethiopia case study. Rev Sci Tech. (2024) 43:115–25. doi: 10.20506/rst.43.3524, PMID: 39222105

[55] Li Y Mayberry D Rushton J. Estimating livestock biomass across diverse populations and data ecosystems. Rev Sci Tech. (2024) 43:23–9. doi: 10.20506/rst.43.3514, PMID: 39222115

[56] Dogonyaro BB van Heerden H Potts AD Fasina FO Casanovas-Massana A Kolo FB . Molecular characterization of Leptospira species detected in the kidneys of slaughtered livestock in abattoirs in Gauteng Province, South Africa. Pathogens. (2023) 12:666. doi: 10.3390/pathogens12050666 37242336 PMC10223745

[57] Supervie V Costagliola D. How was the French BSE epidemic underestimated? C R Biol. (2006) 329:106–16. doi: 10.1016/j.crvi.2005.12.004, PMID: 16439340

[58] Green DM Kao RR. Data quality of the cattle tracing system in Great Britain. Vet Rec. (2007) 161:439–43. doi: 10.1136/vr.161.13.439, PMID: 17906224

[59] Li Y Mayberry D Jemberu W Schrobback P Herrero M Chaters G . Characterizing Ethiopian cattle production systems for disease burden analysis. Front Vet Sci. (2023) 10:1233474. doi: 10.3389/fvets.2023.1233474, PMID: 37885617 PMC10598381

[60] Gilbert W Marsh TL Chaters G Jemberu WT Bruce M Steeneveld W . Quantifying cost of disease in livestock: a new metric for the global burden of animal diseases. Lancet Planet Health. (2024) 8:e309–17. doi: 10.1016/S2542-5196(24)00047-0, PMID: 38729670 PMC11636736

[61] Burgman M. Risks and decisions for conservation and environmental management. Cambridge: Cambridge University Press (2005).

[62] Jemberu W. Estimating disease burden in small ruminants in Ethiopia: Application of the global burden of animal diseases (GBADS) framework. Toulouse: Society for Veterinary Epidemiology and Preventive Medicine (2023).

[63] Saltelli A Ratto M Andres T Campolongo F Cariboni J Gatelli D . Global sensitivity analysis: The primer. Chichester, West Sussex: Wiley (2007).

[64] James LP Salomon JA Buckee CO Menzies NA. The use and misuse of mathematical modeling for infectious disease policymaking: lessons for the COVID-19 pandemic. Med Decis Mak. (2021) 41:379–85. doi: 10.1177/0272989X21990391, PMID: 33535889 PMC7862917

[65] Fletcher D. Model averaging. Springer briefs in Statistics. (2019).

[66] Morgan MG Henrion M. Uncertainty: A guide to dealing with uncertainty in quantitative risk and policy analysis. Cambridge University Press: Cambridge (1990).

[67] Burns PB Rohrich RJ Chung KC. The levels of evidence and their role in evidence-based medicine. Plast Reconstr Surg. (2011) 128:305–10. doi: 10.1097/PRS.0b013e318219c171, PMID: 21701348 PMC3124652

[68] Gardner MJ Altman DG. Confidence intervals rather than P values: estimation rather than hypothesis testing. Br Med J. (1986) 292:746–50. doi: 10.1136/bmj.292.6522.746, PMID: 3082422 PMC1339793

[69] Larkins A Temesgen W Chaters G Di Bari C Kwok S Knight-Jones T . Attributing Ethiopian animal health losses to high-level causes using expert elicitation. Prev Vet Med. (2023) 221:106077. doi: 10.1016/j.prevetmed.2023.106077, PMID: 37976968

[70] Global Burden of Animal Diseases. Scientific & Technical Review World organisation for animal health. Paris (2024) 43. doi: 10.20506/rst.vol.43.3511

[71] Devleesschauwer B Haagsma JA Mangen M-JJ Lake RJ Havelaar AH. The global burden of foodborne disease In: Roberts T, editor. Food safety economics: Incentives for a safer food supply. Springer International Publishing: Cham (2018)

[72] Murray CJL. The global burden of disease study at 30 years. Nat Med. (2022) 28:2019–26. doi: 10.1038/s41591-022-01990-1, PMID: 36216939

[73] Horigan V Simons R Kavanagh K Kelly L. A review of qualitative risk assessment in animal health: suggestions for best practice. Front Vet Sci. (2023) 10:1102131. doi: 10.3389/fvets.2023.1102131, PMID: 36825234 PMC9941190

[74] Brooks-Pollock E de Jong MCM Keeling MJ Klinkenberg D Wood JLN. Eight challenges in modelling infectious livestock diseases. Epidemics. (2015) 10:1–5. doi: 10.1016/j.epidem.2014.08.005, PMID: 25843373

[75] Scoones I. “What is uncertainty and why does it matter?” in STEPS working papers, number 105. The Institute of Development Studies. (2019).

[76] Murungi T Kunihira I Oyella P Mugerwa M Gift P Aceng MJ . The role of religious leaders on the use of HIV/AIDS prevention strategies among young people (15-24) in lira district, Uganda. PLoS One. (2022) 17:e0276801. doi: 10.1371/journal.pone.0276801, PMID: 36301999 PMC9612556

[77] Yibeltal K Workneh F Melesse H Wolde H Kidane WT Berhane Y . God protects us from death through faith and science’: a qualitative study on the role of faith leaders in combating the COVID-19 pandemic and in building COVID-19 vaccine trust in Addis Ababa, Ethiopia. BMJ Open. (2024) 14:e071566. doi: 10.1136/bmjopen-2023-071566, PMID: 38653509 PMC11043698

[78] Wilkinson MD Dumontier M Aalbersberg IJ Appleton G Axton M Baak A . The FAIR Guiding Principles for scientific data management and stewardship. Sci Data. (2016) 3:160018. doi: 10.1038/sdata.2016.18 26978244 PMC4792175

[79] Hanea AM McBride MF Burgman MA Wintle BC Fidler F Flander L . Investigate discuss estimate aggregate for structured expert judgement. Int. J. Forecast. (2017) 33, 267–279. doi: 10.1016/j.ijforecast.2016.02.008

